# Management and Clinical Outcomes of Pancreaticoduodenal Injuries at a Tertiary Care Centre in Southern India

**DOI:** 10.7759/cureus.84027

**Published:** 2025-05-13

**Authors:** Lokeshwaran Ayyasamy, Senthilkumaran Govindaraj Raman, Sudhagar Rengasamy, Arun Raja Alagendran, Vivek G Nath, Mathews Micheal, M Pon Chidambaram, U Aravindan

**Affiliations:** 1 Department of Surgical Gastroenterology & GI Oncology, Thanjavur Medical College, Thanjavur, IND

**Keywords:** blunt trauma, duodenal injuries, morbidity, mortality, pancreatic injuries

## Abstract

Objective: Pancreaticoduodenal injuries are complex, and early diagnosis is often missed due to their retroperitoneal location. Serial clinical monitoring and imaging are essential for the management of these injuries. Morbidity and mortality increase in patients with delayed presentation. The aim of this study is to analyse the demography, mode of presentation, grading, management, morbidity and mortality of these injuries at our centre.

Methods: This is an ambispective study conducted on 31 patients over a period of five years, between October 1, 2019 and October 15, 2024.

Results: Most patients were male (93.5%). Blunt trauma was associated with the majority of cases (83.8%). Twenty-nine patients were hemodynamically stable on arrival at the trauma/emergency ward. Postoperative morbidity was around 32% in our study, whereas mortality was approximately 7%. Nonoperative management was followed in five cases of high-grade (Grade 4) pancreatic injury. One patient with Grade 5 pancreaticoduodenal injury underwent an emergency Whipple procedure. In our study, postoperative morbidity associated with delayed admission beyond 48 hours from the time of injury was statistically significant (p = 0.029).

Conclusion: A high index of suspicion is needed in every case. Delayed diagnosis and treatment negatively impact morbidity and mortality. In selected cases of high-grade pancreatic injuries, conservative management with serial follow-up and imaging can be considered at a well-equipped tertiary care centre.

## Introduction

Pancreaticoduodenal injuries are rare, occurring in about 1-5% of abdominal trauma cases in India. The anatomical location makes these injuries complex, and their clinical presentation makes early diagnosis difficult. Serial clinical monitoring is needed in every case [1-5]. The most common mechanism of injury is blunt trauma, seen in 60%-80% of cases, followed by penetrating trauma in pancreatic injuries (20%), mostly due to stab injuries or gunshot wounds [3]. Most of these injuries are hemodynamically stable unless associated with other organ injuries. Associated organ injuries pose a serious threat to survival and increase morbidity [4,5].

Triple-phase CT is the imaging modality of choice for diagnosing these injuries. Major pancreatic duct injuries are also better delineated on triple-phase CT of the abdomen [3-6]. Contrast CT is done after adequate resuscitation of the injured patient. Similarly, examination of other systems is also necessary to rule out associated injuries. The Advanced Trauma Life Support protocol should be followed for all trauma cases [7].

Early diagnosis and treatment play a major role in management. With delayed diagnosis and treatment, morbidity may rise to 36%-60% [2,4,5], and mortality up to 18%-23% [4,5]. The risk of complications like fistula, abscess or bleeding increases in cases with delayed diagnosis [4]. The most common cause of early death in pancreaticoduodenal injuries is exsanguination from associated injuries due to haemorrhagic shock. Late deaths are usually due to sepsis or multiple organ failure [8].

## Materials and methods

This ambispective study was conducted at Thanjavur Medical College and Hospital. The study period was between October 1, 2019 and October 15, 2024. A total of 31 patients were included in the study. The study was approved by the Institutional Ethical Committee of Thanjavur Medical College (IEC approval no: 675/2022). Informed written consent was obtained from all participants.

Inclusion criteria

Patients admitted to the trauma ward and diagnosed with pancreaticoduodenal injuries were included. Patients referred from peripheral centres or outside hospitals with confirmed diagnoses of these injuries were also included.

Exclusion criteria

Patients with associated major vascular injuries and other solid organ injuries (liver, spleen, kidney) were excluded from the study.

Collected data

The following data were collected: age, sex, duration between injury and time of presentation, mode of injury, haemodynamic status, diagnostic modality, type of injury, grade of injury, associated injuries, management, hospital stay, postoperative complications, morbidity and mortality.

Statistical analysis

The collected data were entered in Microsoft Excel 2016 (Microsoft Corporation, Redmond, USA) and analysed using IBM SPSS Statistics for Windows, Version 29 (Released 2023; IBM Corp., Armonk, New York, United States). Data were expressed as n (%) and mean (SD) for categorical and continuous variables, respectively. The unpaired t-test was used to compare means between two groups. Fisher’s exact test was used to compare frequencies between groups. A p-value < 0.05 was considered statistically significant.

## Results

Demography

Most of the patients were male (N = 29, 93.5%), with over half aged 21-40 years (N = 16, 51.6%), commonly due to road traffic accidents, as shown in Table 1.

**Table 1:** Demographic details of patients with pancreaticoduodenal injuries (N = 31) Data are expressed as N (%). Total N = 31

S. No.	Parameter		N	%
1	Gender	Men	29	93.5
		Women	2	6.5
2	Age category	≤20 years	6	19.4
		21–30 years	9	29
		31–40 years	7	22.6
		41–50 years	6	19.4
		51–60 years	3	9.6

Mechanism and type of injuries

The most common mechanism of injury was blunt abdominal trauma (N = 26, 83.8%), mostly due to road traffic accidents or falls from height (Table 2). In our study, 20 patients (64.5%) had pancreatic injuries and 11 patients (35.5%) had duodenal injuries (Table 2).

**Table 2:** Description of various clinical characteristics of patients with pancreaticoduodenal injuries (N=31) Data are expressed as N(%).

S.No	Parameter		N	%
1	Mechanism of injury (N=31)	Blunt trauma	26	83.8
		Penetrating trauma	5	16.2
2	Type of injury (N=31)	Duodenal	11	35.5
		Pancreatic	20	64.5
3	Grading of duodenal injuries (N=11)	Grade 2	1	9.1
		Grade 3	8	72.8
		Grade 4	2	18.1
4	Grading of pancreatic injuries (N=20)	Grade 2	3	15
		Grade 3	10	50
		Grade 4	6	30
		Grade 5	1	5

Grading of injuries

Grading of injuries was based on the AAST guidelines [3]. The most common pancreatic injury in our study was Grade 3 (N = 10, 50%), followed by Grade 4 (N = 6, 30%), Grade 2 (N = 3, 15%) and Grade 5 (N = 1, 5%) (Table 2). Among duodenal injuries, Grade 3 (N= 8, 72.8%) was most frequently observed (Table 2).

Admission from injury and haemodynamic status

With respect to the time of presentation from injury, 16 (52%) patients presented after more than 48 hours (late admission), whereas 15 (48%) patients presented within 48 hours (early admission). Most patients were haemodynamically stable (N = 29, 94%). Two patients who presented to our hospital more than 48 hours after injury were hemodynamically unstable and required resuscitation and emergency laparotomy, which revealed a Grade 4 duodenal injury.

Management of pancreaticoduodenal injuries

**Figure 1:**
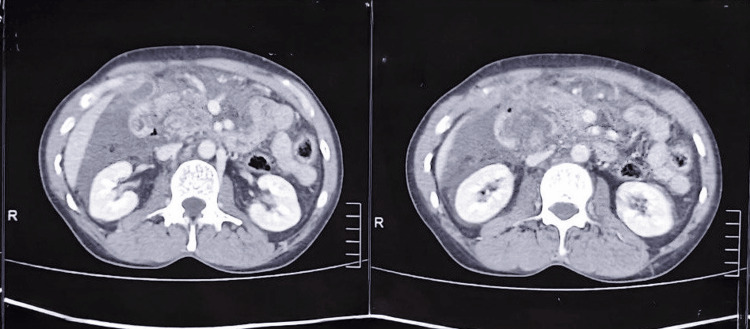
Grade 5 pancreatic injury in contrast CT abdomen

**Figure 2:**
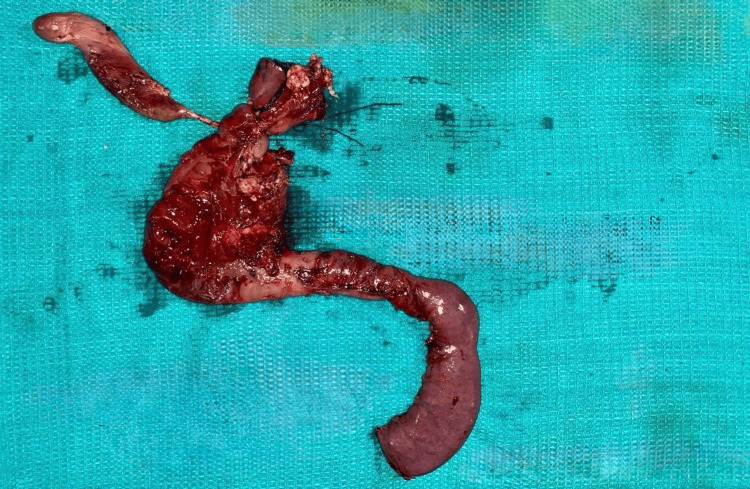
Emergency Whipple procedure for Grade 5 pancreatic injury

**Figure 3:**
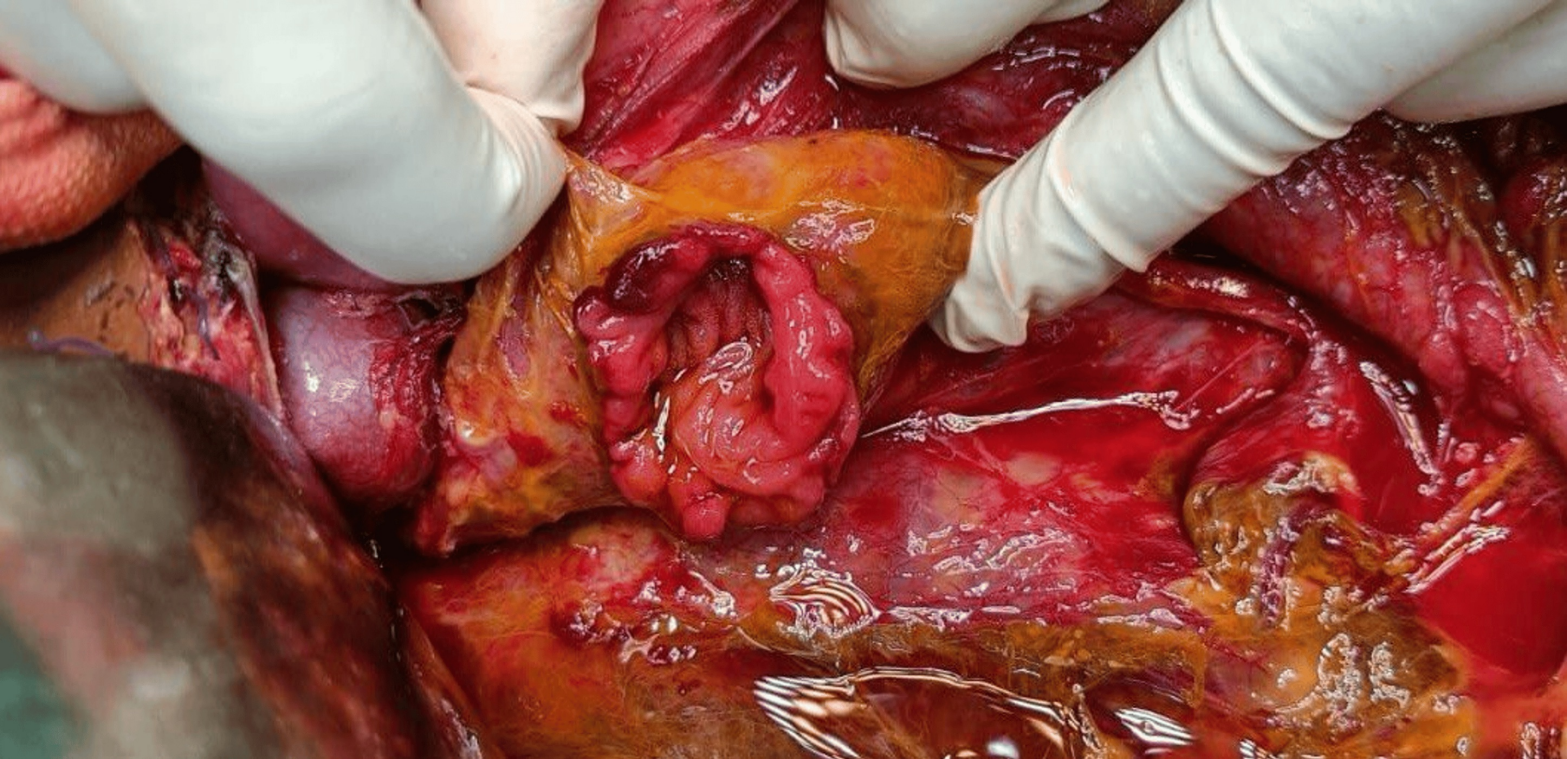
Grade 3 duodenal injury

**Figure 4:**
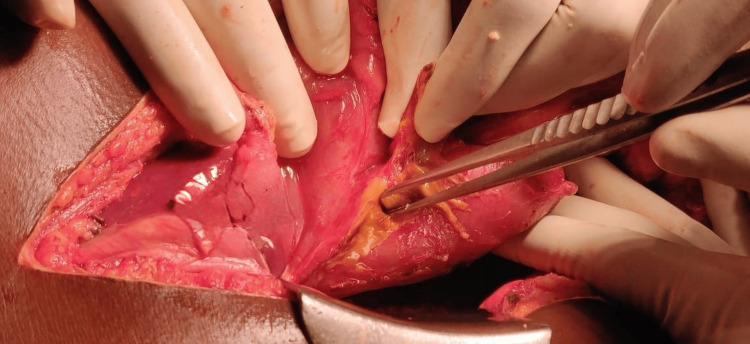
Grade 2 duodenal injury

Most of the Grade 3 pancreatic injuries were treated with distal pancreatectomy and splenectomy (N = 7, 35%). Two patients (10%) underwent spleen-preserving distal pancreatectomy (Table 3). One Grade 5 (5%) pancreatic injury required an emergency Whipple procedure, as shown in Figures 1, 2. All patients with duodenal injuries (Figures 3, 4) underwent surgical management. Six patients (55%) underwent primary closure with pyloric exclusion.

**Table 3:** Management of pancreatic injuries Data are expressed as N (%). Total N = 20

Management	Pancreatic Injuries (N = 20)
Distal pancreatectomy and splenectomy	7 (35%)
Spleen-preserving distal pancreatectomy	2 (10%)
Laparotomy with debridement and lavage	3 (15%)
Trauma Whipple	1 (5%)
Nonoperative management	7 (35%)

Nonoperative management

**Figure 5:**
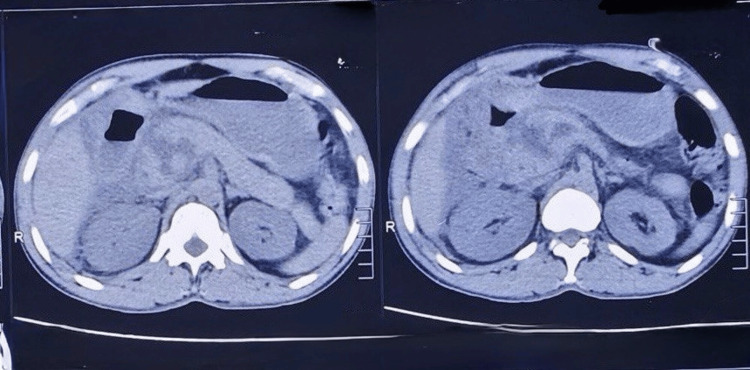
CT abdomen showing Grade 4 pancreatic injury

**Figure 6:**
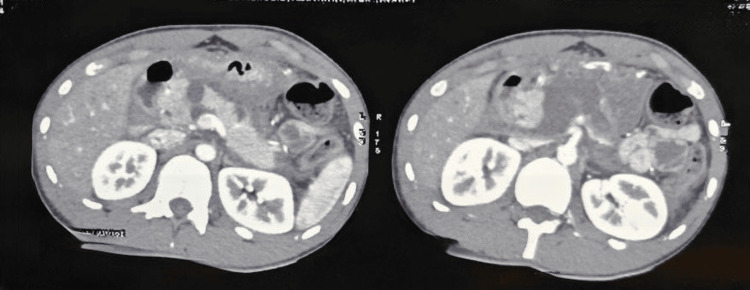
Grade 4 pancreatic injury follow-up after three months

Seven patients with pancreatic injuries (35%) were managed nonoperatively. Three cases of Grade 4 pancreatic injury with stable hemodynamic status and no signs of clinical sepsis (tachycardia, peritonitis, or elevated total leukocyte count), along with localized collections seen on imaging, were managed conservatively. Follow-up imaging (triple-phase CT and MRCP) revealed pseudocysts that were asymptomatic, and these patients were kept under observation (Figures 5, 6). Two patients with Grade 4 pancreatic injury, presenting with localized collections and clinical sepsis (fever, tachycardia), were managed with pigtail catheterization alone. Two cases of Grade 2 pancreatic injury were also treated conservatively.

Postoperative morbidity and mortality

Patients who had late admission (>48 h) or delayed presentation to the hospital experienced increased complications such as bile leak and pancreatic fistula, which led to higher reintervention rates. The overall postoperative morbidity was 32% (Table 4), and it was statistically significant in the late admission group (p = 0.029). Mortality occurred in two patients with Grade 4 duodenal injuries (7%), primarily due to sepsis. The duration of hospital stay was also significantly longer in the morbidity group (p < 0.0001), as shown in Table 5.

**Table 4:** Comparison of type of morbidity, mortality and overall morbidity with respect to the time of admission in patients with pancreaticoduodenal injuries observed in the study Data are expressed as n (%). Fisher’s exact test was used to compare frequencies between the two groups. *indicates p <0.05 and considered statistically significant. NS: Not significant

S.No	Parameters		Admission <48 hrs of injury (N=15)		Admission ≥48 hrs of injury (N=16)		Fisher exact value	df	p-value
			n	%	n	%			
1	Type of morbidity	Biliary fistula	1	6.6	3	20	1.381	4	0.847 (NS)
		Pancreatic fistula	2	13	5	31			
		Pigtail drainage	1	6.6	3	20			
		Relaparotomy	0	0	2	12.5			
2	Mortality	Present	0	0	2	12.5	2.004	1	0.157 (NS)
		Absent	15	100	14	87.5			
3	Morbidity	Present	2	13.3	8	50	4.763	1	0.029*
		Absent	13	86.7	8	50			

**Table 5:** Comparison of the duration of hospital stay (days) with respect to morbidity status observed in patients with pancreaticoduodenal injuries Data are expressed as mean with standard deviation. An unpaired t-test was used to compare the means between two groups. *indicates p<0.05 and considered statistically significant.

S.No	Parameters	Morbidity present (N=10)		Morbidity absent (N=21)		T value	df	p-value
		Mean	SD	Mean	SD			
1	Duration of hospital stay (days)	15	6	8	3	4.371	29	<0.0001*

## Discussion

In our study, there were 31 patients diagnosed with duodenal (N = 11, 35%) and pancreatic injuries (N = 20, 65%). Of the total abdominal trauma cases (N = 530) in our study, the incidence of pancreaticoduodenal injuries was 5.8%, which was comparable to the incidence reported in a study done by Bavishi et al. [9].

The most common age group was 20-40 years. Young male patients were affected more frequently, as they are more involved in daily physical activities and also have an alleged history of abusive behaviour.

The most common mechanism of injury was blunt trauma (84%) in our study, mostly due to road traffic accidents, whereas in studies done by Menahem et al. [10] and Pata et al. [11], it was about 60%-80%. Similarly, the cause of penetrating trauma (16%) in our study was stab injury and bull gore injury, rather than gunshot wounds, which is more common in South India due to social circumstances and the presence of a large farming community in and around the region.

The sensitivity of triple-phase CT in diagnosing these injuries was 85%, which was similar to the study conducted by Lahiri et al. [12], who reported 80% sensitivity, especially in emergency settings. MRCP was done in doubtful cases when needed.

According to the AAST guidelines [3], Grade 3 pancreatic injuries were seen in 10 (50%) cases, Grade 2 in three cases (15%), Grade 4 in six cases (30%) and Grade 5 in one case (5%). Similarly, Grade 3 duodenal injuries were seen in eight patients (73%) and Grade 2 in three patients (27%).

Operative management was performed in all cases of duodenal injury. The severity of the injuries, particularly Grade 4 and Grade 5, influenced surgical decision-making, often necessitating damage control surgery and contributing to increased morbidity and mortality. The most common procedure (n = 6) performed for Grade 3 duodenal injuries was primary closure with pyloric exclusion, along with antecolic gastrojejunostomy and feeding jejunostomy. The role of pyloric exclusion remains debatable in many studies [13].

High-grade injuries, such as Grade 4 pancreatic injuries, were managed conservatively based on hemodynamic status, imaging findings, the presence or absence of clinical sepsis, and the absence of other abdominal injuries requiring surgery. Approximately five cases of Grade 4 pancreatic injury in adults and two cases of Grade 2 pancreatic injury in children (with higher success in conservative management in children) were managed non-operatively, as discussed in the study by Mansiroglu et al. [14].

Grade 3 pancreatic injuries were mostly managed with distal pancreatectomy and splenectomy, as several patients failed conservative management (clinical sepsis, failure of endoscopic and percutaneous interventions). In one case, early presentation allowed for a spleen-preserving procedure due to minimal contamination and reduced inflammatory adhesions. This technical approach was described in the study by Mulpuri et al. [15]. One case of Grade 5 pancreatic injury required an emergency Whipple procedure rather than a staged operation (damage control surgery) because of stable hemodynamic status (absence of hypothermia, acidosis, and coagulopathy) [16].

Patients with late admission (> 48 hours) experienced increased postoperative complications such as bile leak, pancreatic fistula and relaparotomy, compared to those admitted early (< 48 hours). Morbidity in the late admission group was statistically significant (chi-square test, p = 0.029). The overall morbidity was 32%, and the mortality (N = 2/31) was 7%, mainly due to sepsis.

Limitations of the study

Pancreaticoduodenal injuries are rare and less common than other abdominal injuries. This rarity makes it difficult to establish clear guidelines for both diagnosis and treatment. The complexity of managing these injuries presents significant challenges, particularly in determining the appropriate timing and extent of surgical intervention. Treatment strategies continue to evolve, which hampers the ability to conduct long-term studies and follow up with patients across different therapeutic approaches.

## Conclusions

A high index of suspicion is needed in every case. Delayed diagnosis and treatment adversely impact morbidity and mortality. In selected cases of high-grade pancreatic injuries, conservative management can be considered with serial follow-up and imaging in a well-equipped tertiary care centre. The role of conservative management in Grade 4 pancreatic injury depends on the haemodynamic status and clinical condition (with attention to signs of sepsis). Patients may require intervention with either pigtail drainage or minimally invasive surgery (lavage and drainage), depending on the location of collections in these injuries. Trauma Whipple is a morbid procedure and can be performed only in selected stable cases. Therefore, prompt early referral is key to managing these injuries effectively. Timely transfer to a well-equipped tertiary care centre with full hepato-pancreato-biliary expertise is essential to reduce morbidity and mortality.
